# Microbiome‐Based Assessment of Topical Scalp Product‐Associated Microbiome Modulation

**DOI:** 10.1111/jocd.71238

**Published:** 2026-10-28

**Authors:** Uyen Thy Nguyen, Joseph Skarlupka, Sofia Iglesia, Tatiana Kononov, Alisar Zahr, Elsa Jungman, Haryung Grace Park

**Affiliations:** ^1^ HelloBiome, ELSI Skin Health Inc. San Francisco California USA; ^2^ Revision Skincare Dallas Texas USA

## Abstract

**Background:**

The scalp microbiome plays a crucial role in maintaining scalp health, yet few studies have examined how topical scalp cosmeceuticals affect bacterial and fungal communities. In this study we aim to investigate the impact of a scalp serum on the scalp microbiome and physiology.

**Methods:**

In this 12‐week IRB‐approved study, changes in the scalp microbiome before, during, and after the use of a scalp serum were measured in 56 participants. The bacterial and fungal microbiomes were assessed using 16S rRNA and ITS2 amplicon sequencing and analyzed key scalp parameters such as scalp thickness, intensity, moisture and pH.

**Results:**

We identified a significant increase in the bacteria‐to‐fungi load ratio after 4 and 12 weeks of product use relative to baseline, driven primarily by increased bacterial load. Fungal Shannon diversity also increased after 4 and 12 weeks of product use relative to baseline, while beta‐diversity analysis showed a significant shift in bacterial composition. Pairwise comparisons of the dominant taxa identified significant increases in *Cutibacterium namnetense* and *Cladosporium* sp. abundances, along with a significant increases in other low‐abundance species after 12 weeks of product use. Spearman's correlation testing identified a significant negative correlation between *Malassezia sympodialis* and scalp thickness, with additional nonsignificant trends linking microbial composition to hydration and pH.

**Conclusion:**

In conclusion, this study suggests that topical treatments may support scalp health by respecting the balance of the scalp microbiome.

## 1 Introduction

A growing body of research has highlighted the connections between the scalp microbiome and hair and skin health. The scalp microbiome is dominated by *Cutibacterium* and *Staphylococcus*, with *Malassezia* as the dominant fungal genus [1]. While *Malassezia restricta* has been implicated in dandruff through its lipase activity and inflammatory metabolites [2, 3, 4], it is also abundant on healthy scalps. Similarly, seborrheic dermatitis is associated with reduced bacterial diversity and *Malassezia* overgrowth [5], often accompanied by a decline in *Cutibacterium acnes*. These findings underscore that scalp conditions reflect disruptions in microbial community balance rather than the presence of specific microbes [6, 7].

Growing interest in scalp health has prompted researchers to investigate how hair care products affect the scalp microbiome [8]. Despite the wide range of products marketed for moisturizing, oil control, and dandruff treatment, their impacts on the scalp microbiome remain poorly explored [9]. Emerging studies have demonstrated that certain antifungal shampoos can reduce *Malassezia* abundance but may also disrupt beneficial microbial populations, while some plant‐based formulations have shown promising selective modulations in microbial communities [10].

In this open‐label clinical study, we investigated the impact of a scalp serum on the scalp microbiome in 56 participants over a 12‐week period. Microbial composition was compared to baseline profiles to evaluate intrapersonal shifts and measure the effects of the serum on the scalp microbial community. In parallel, we analyzed correlations between microbial abundance and key scalp physiological parameters, including thickness, hydration, pH, and microcirculation.

Our findings provide new evidence that topical treatments can support scalp health by promoting a balanced microbial community. Moreover, they demonstrate the feasibility of developing innovative topicals that specifically modulate the scalp microbiome through various formulation strategies, opening new avenues for creating efficacious products that address common scalp conditions through microbiome‐targeted approaches.

## 2 Methods

### 2.1 Participant Recruitment Criteria

Participants in the study were recruited in accordance with the Institutional Review Board (IRB) approval number [11598]. This open‐label 12‐week study recruited a total of 60 female participants aged 25–65 with self‐perceived mild to moderate hair thinning and a Ludwig score of I‐1 to I‐4 assessed by a board‐certified dermatologist, and willingness to refrain from hair dyeing for the duration of the study period. Exclusion criteria included significant hair loss (Ludwig ≥ II‐1), severe scalp conditions, active scalp disease or alopecia, colored or recently dyed hair, hair extensions or tight hairstyles, and use of medicated scalp products (12 months), hair growth/thinning treatments (6 months), hair vitamins (3 months), or hormone replacement therapy. Pregnant or breastfeeding individuals were also excluded. Fifty‐six out of 60 participants had fully completed scalp microbiome sampling.

### 2.2 Product Usage

A scalp serum was formulated as a lightweight water‐based serum with botanical extracts, plant‐derived extracellular vesicles, caffeine, fermented yerba mate, a biomimetic peptide, plant adaptogens, antioxidants including resveratrol and rosemary extract, and prebiotic and postbiotic technology.

Over 12 weeks, the scalp serum was applied once a day to wet or dry hair according to the instructions provided. In addition, participants were given a choice between three different shampoo/conditioner sets (TRESemme Flawless Curls, Suave Lush & Coily, or Suave Rose Oil Infusion) based on their hair type. Participants used these products three times weekly, beginning 7 days prior to the start date as a wash‐out period and continuing throughout the study, during which all other scalp and hair products were discontinued.

### 2.3 Microbiome Sample Collection

Microbiome samples were self‐collected from the frontal scalp area using sterile swabs at three different time points: T1 (baseline), T2 (4 weeks), and T3 (12 weeks). Before swabbing, participants followed a 24‐h wash‐out period, refraining from scalp and hair products, styling devices, scalp washing, and strenuous activity. Swabbing was performed by parting the hairline at the midline and swabbing the frontal scalp for 60 s. Samples were stored in DNA/RNA Shield buffer and shipped within 4 days.

### 2.4 Metadata Collection

Participants completed a self‐reported questionnaire covering demographics, scalp and skin profiles, and lifestyle habits. Scalp thickness and intensity were measured by ultrasound, while pH, moisture, and blood circulation were assessed via an instrument.

### 2.5 Microbiome Sequencing and Statistical Analysis

Microbial DNA was extracted from scalp swabs and sequenced at the 16S rRNA V1–V3 region for bacteria and the ITS2 region for fungi. Contaminants were removed using a proprietary algorithm, and all processed samples yielded high‐quality reads. Raw sequencing data were transformed into relative abundance and analyzed in R (v 4.3.3) and RStudio using https://www.r‐project.org/ phyloseq (v1.46.0) [11] for preprocessing, and MicroViz (v0.12.6) [12] and ggplot2 (v3.5.1) for visualizations. The Wilcoxon signed‐rank test was used for pairwise comparisons, and community‐level differences were assessed by PERMANOVA on Bray–Curtis dissimilarity. Statistical significance was defined as an adjusted *p* < 0.05 following Benjamini–Hochberg and Holm corrections.

### 2.6 Privacy and Cybersecurity

All personally identifiable information (PII) was removed before microbiome analyses, which were conducted locally on encrypted hardware storage using FileVault (Apple) and BitLocker (Microsoft). For data transfers via servers, we employed secure HTTPS connections with current SHA‐256 and RSA encryption protocols. Two‐factor authentication was mandatory to access critical team resources, including GitHub, AWS, and OneDrive throughout the study.

## 3 Results

### 3.1 Demographic Characteristics of Study Population

Fifty‐six female participants completed the full study. Participants were evenly distributed across ages from their 20s to 60s (Table 1), and were White or Caucasian (71.4%), with smaller proportions of Black or African American (16.1%), Asian (7.1%), Hispanic or of Spanish origin (1.8%), and multi‐ethnic or other (3.6%). Hair types were predominantly wavy (44.6%) or straight (23.2%), with fewer curly (21.4%), kinky (8.9%), and coily (1.8%). Scalp types ranged in balanced (46.4%), dry (26.8%), and oily (5.4%) (Table 1).

**TABLE 1:** Demographic distribution of participants including age, ethnicity, hair type, and scalp type.

	Age distribution					
	20s (*n* = 9)	30s (*n* = 8)	40s (*n* = 11)	50s (*n* = 17)	60s (*n* = 11)	Total (*n* = 56)
Ethnicity						
Asian	11.1	25.0	0.0	5.9	0.0	7.1
Black or African American	22.2	25.0	9.1	17.6	9.1	16.1
Black or African American White or Caucasian	11.1	0.0	0.0	0.0	0.0	1.8
Hispanic or of Spanish origin, Latinx	0.0	0.0	0.0	5.9	0.0	1.8
Other	0.0	12.5	0.0	0.0	0.0	1.8
White or Caucasian	55.6	37.5	90.9	70.6	90.9	71.4
Hair type						
Coily	0.0	0.0	0.0	0.0	9.1	1.8
Curly	22.2	12.5	27.3	23.5	18.2	21.4
Kinky	22.2	25.0	9.1	0.0	0.0	8.9
Straight	33.3	37.5	36.4	11.8	9.1	23.2
Wavy	22.2	25.0	27.3	64.7	63.6	44.6
Scalp type						
Balanced	44.4	37.5	54.5	41.2	54.5	46.4
Dry	44.4	37.5	27.3	23.5	9.1	26.8
Not sure	0.0	25.0	18.2	29.4	27.3	21.4
Oily	11.1	0.0	0.0	5.9	9.1	5.4

### 3.2 Quantification of Scalp Microbial Abundance

To determine the overall shift in the scalp microbiome, we calculated the load ratio by dividing total bacteria load by fungal microbial load. We found a significant increase in the bacteria‐to‐fungi load ratio after T2 (*p* < 0.001) and T3 (*p* < 0.001) relative to baseline (Figure 1a). Total bacterial load significantly increased throughout the product use (Figure 1b). While total fungal load did not change after 4 weeks, prolonged usage significantly increased fungal load compared to 4 weeks (*p* < 0.05). However, in comparison to baseline, there was no significant difference (Figure 1c). This demonstrates that the shift in bacteria‐to‐fungi load ratio is driven by changes in the bacterial community.

**FIGURE 1:**
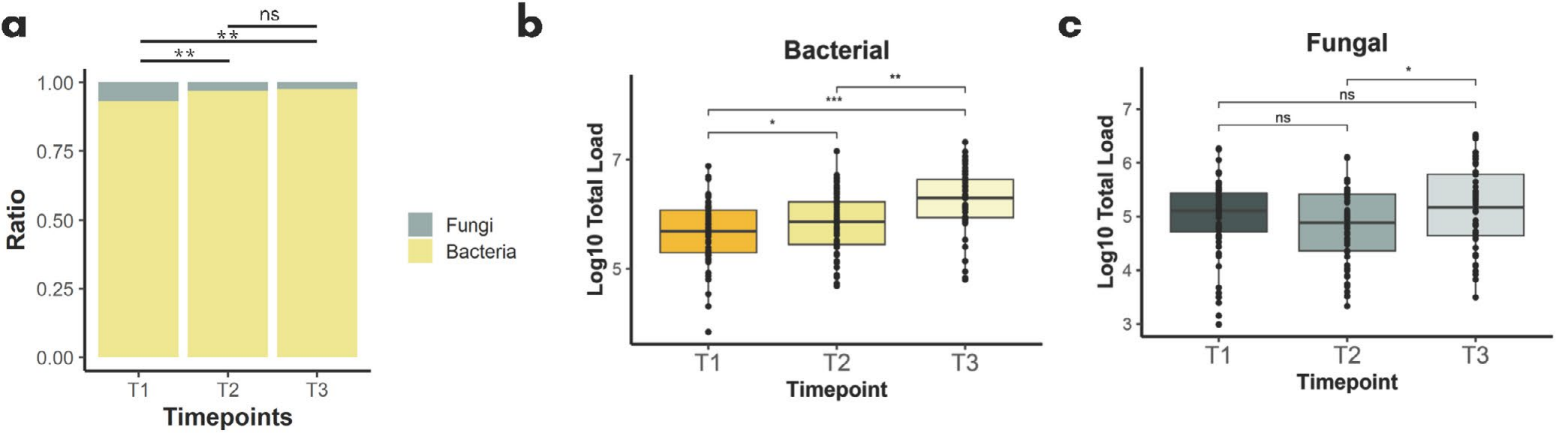
Temporal dynamics in microbial abundances. (a) Bacterial to fungal load ratio. Individual (b) bacterial and (c) fungal load. The *p*‐values were calculated using the Wilcoxon's test and adjusted using Holm's method. *p < 0.05, **p < 0.001, ***p < 0.0001.

### 3.3 Microbial Composition Shifts Following Topical Serum Application

We then calculated the Shannon diversity index, capturing both the number of species present (richness) and their distribution (evenness). While there were no shifts in the bacterial community (Figure 2a), fungal diversity significantly increased from baseline at both time points, T2 (*p* < 0.001) and T3 (*p* < 0.001) (Figure 2b). We calculated Bray–Curtis dissimilarity to measure overall community structure, revealing a significant shift in bacterial composition, driven by high variability in T3 samples (Figure 2c). In contrast, the fungal composition remained stable over the course of 12 weeks (Figure 2d). Together, these findings reveal that prolonged use of the scalp serum significantly shifted the bacterial community composition while preserving fungal community structure.

**FIGURE 2:**
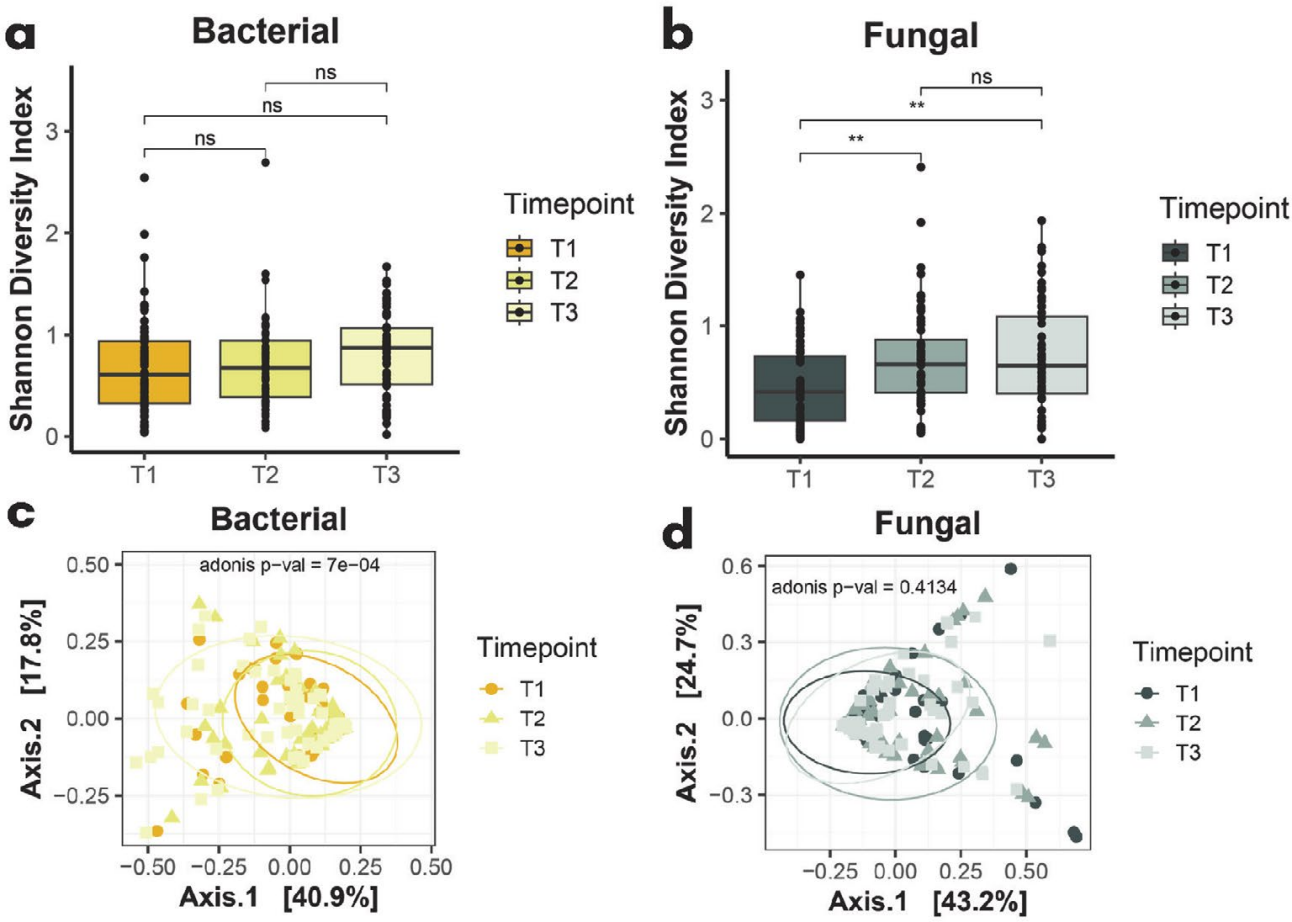
Microbial composition assessment across time. Shannon diversity index for (a) bacterial and (b) fungal communities. The *p*‐values were calculated using the Wilcoxon's test and adjusted using Holm's method. **p < 0.01. Community structure for (c) bacteria and (d) fungi using Bray–Curtis dissimilarity PCoA. PERMANOVA was used to measure statistical differences between timepoints with the ADONIS *p*‐value indicating significance of group separation.

### 3.4 Microbial Correlations With Clinical Scalp Parameters

As bacterial composition and fungal diversity showed pronounced changes, we examined the top 10 bacterial and fungal species by averaging relative abundances per time point. The top bacterial species belong to four different genera: *Corynebaterium, Cutibacterium, Lawsonella, Staphylococcus* (Figure 3a), which are commensals often found on a healthy scalp [10, 13, 14]. A pairwise comparison of the relative abundances of the top 10 species revealed that *C*. *namnetense* significantly increased after 12 weeks of product use compared to baseline samples (*p* < 0.0001) across most participants (Figure 3a; Table S1). Notably, *C. namnetense* was undetectable in all participants at baseline, whereas after 12 weeks of product use, it was detected in 36 participants (64.29% prevalence), with a mean relative abundance of 17.67% among those participants (Table S2). This suggests that the extended product use may have driven colonization by *C. namnetense* on the scalp. Due to this shift, *C. acnes* showed a decreasing tendency after 12 weeks of product use compared to baseline. Subsequently, we calculated Spearman's correlation between the relative abundance of the top species integrated with ultrasound (intensity and scalp thickness in mm) and other scalp instrumental results (pH, moisture meter, and blood circulation). While correlations were not significant, we found a positive correlation between *C. namnetense* and ultrasound intensity (Figure 3b), suggesting that this species is possibly linked to improved scalp structural integrity. A positive correlation between *Staphylococcus hominis* and moisture level was observed (Figure 3b), indicating that this commensal species thrives in more hydrated scalp environments where it may contribute to microbial community stability.

**FIGURE 3:**
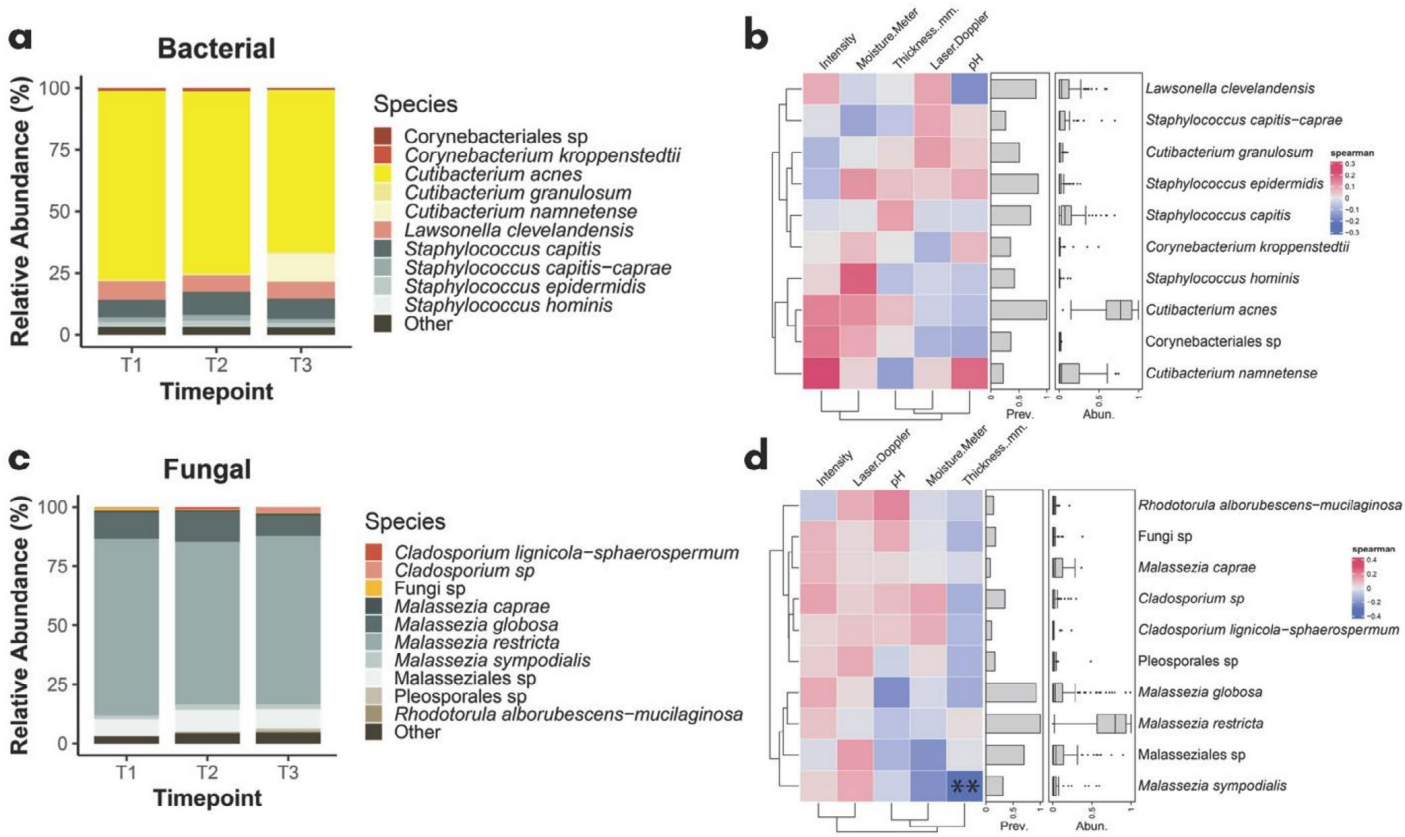
Average abundance of the top 10 species (a, c) and their correlations with ultrasound (intensity and scalp thickness in mm) and instrumental (pH, moisture meter, and laser doppler) data (b, d). Spearman's correlation was used along with Holm's adjustment method. Cell labels indicate correlation coefficients. ** indicates *p* < 0.01.

Examination of the fungal community composition revealed a prominent presence of *Malassezia* species with a lower abundance of *Cladosporium* species and other transient fungal species. *Cladosporium* sp. significantly increased after 12 weeks of product use compared to baseline (*p* < 0.001), while other low abundance species also significantly increased after prolonged product use (Figure 3c; Table S3). Spearman's correlation coefficient between *M. sympodialis* and scalp thickness demonstrated a significant negative correlation (*p* < 0.01) (Figure 3d), demonstrating that decreased scalp thickness was associated with an increase in *M. sympodialis* abundance. In addition, though not significant, negative correlations were shown between *M. sympodialis* and *Malasseziales* sp. and moisture level, while *Malassezia globosa* was negative correlated with pH level (Figure 3d).

## 4 Discussion

In this study, we demonstrated that daily application of the scalp serum was associated significant positive shifts in the scalp microbiome. Following 4 weeks of product use, total bacterial load increased, whereas fungal load remained stable. Over 60% of participants showed increased Shannon diversity in the fungal community at both follow‐up time points relative to baseline. Compositional analysis revealed significant changes in the bacterial community structure, with increased inter‐individual variation (PERMANOVA, *p < 0.001*). In contrast, the fungal community structure remained stable despite prolonged serum use.

*C. namnetense* abundance increased in over 60% of participants after 12 weeks. The role of *C. namnetense* is not clear, but its lipid profiles are different from other *Cutibacterium* species [15]. While the association between *C. namnetense* and Quinquaud's folliculitis decalvans has been reported [16], significant reductions in *C. namnetense* abundance were observed in lesional scalp areas of psoriasis capitis patients [17]. In our study, *C. namnetense* and *S. hominis* abundances increased and correlated with improved scalp structural integrity and higher moisture levels, indicating that scalp moisture supports both barrier integrity and a beneficial microbial community. Furthermore, the increase in *C. namnetense* abundance was not associated with any adverse symptoms.

Concurrently, *Cladosporium* sp. abundance significantly increased from an average of 0.4%–2.5% across 57% of the participants. A clinical study of an anti‐seborrheic dermatitis shampoo revealed significant increases in *Cladosporium and* other low‐abundance Ascomycota alongside improvements in dandruff, erythema, and pruritus [18]. Notably, *Cladosporium* produces bioactive natural products, including polyketides, alkaloids, steroids, and terpenoids [19], suggesting potential therapeutic relevance beyond its role as a community member.

Correlations between dominant fungal species and various scalp parameters revealed a significant association between *M. sympodialis* abundance and decreased scalp thickness, consistent with its established role in barrier disruption and inflammation, whereby elevated pH promotes allergen release from *M. sympodialis* [20]. Genomic analyses have further implicated this species in atopic eczema, where sexual reproduction potentially enhances its virulence [21]. Its relative abundance is also substantially elevated on dandruff‐affected scalp compared to healthy scalps [22]. In our study, *M. sympodialis* abundance was less than 3%, a level typically found on a healthy scalp. Importantly, despite this observed correlation, *M. sympodialis* abundance did not change after product use, suggesting the treatment preserved this species within the range observed on healthy scalps.

Traditional antifungal shampoos, such as ketoconazole [23], induce broad‐spectrum disruptions affecting both pathogenic and beneficial microorganisms, while also suppressing sebum production and interfering with *Malassezia* lipid metabolism [24]. A longitudinal study suggests that maintaining beneficial species, particularly *C. acnes* and *M. globosa* , whose abundances negatively correlate with dandruff, is crucial for scalp health [10]. Probiotic‐based approaches have demonstrated that both heat‐killed and live probiotic applications can reduce *Malassezia* and *Staphylococcus* while improving dandruff, sebum secretion, hair growth, and clinical scores [25, 26]. Our scalp serum supports the native bacterial community without introducing exogenous species, promoting bacterial diversification while maintaining fungal stability, a microbiome‐preserving effect consistent with recent findings on a fermentation filtrate‐containing shampoo [27]. In summary, these findings support microbiome modulation as a central strategy for next‐generation scalp care. Targeting beneficial microbiome shifts, rather than broad antimicrobial effects, thus represents a promising development strategy.

## Author Contributions

Conceptualization: Sofia Iglesia, Alizar Zahr, Haryung Grace Park, Elsa Jungman. Data curation: Uyen Thy Nguyen. Formal analysis: Uyen Thy Nguyen. Writing – original draft preparation: Uyen Thy Nguyen, Joseph Skarlupka. Writing – review and editing: Uyen Thy Nguyen, Joseph Skarlupka, Sofia Iglesia, Alisar Zahr, Haryung Grace Park. All authors have read, commented on, and approved the final manuscript.

## Funding

The authors have nothing to report.

## Ethics Statement

All participants provided informed consent, and data confidentiality was maintained throughout the study.

## Consent

Consent was received by all participants.

## Conflicts of Interest

The authors declare no conflicts of interest.

## Supporting information


**Table S1:** Mean relative and absolute (estimated cells per swab) abundances of the top 10 bacterial species across all time points.
**Table S2:** Cutibacterium namnetense prevalence and relative abundance at each time point.
**Table S3:** Mean relative and absolute (estimated cells per swab) abundances of the top 10 fungal species across all time points.

## Data Availability

The data that support the findings of this study are not publicly available due to restrictions imposed by the third‐party sequencing provider. The raw sequencing data were generated and processed by an external sequencing partner and were used under restricted data‐use terms for this study. The authors do not have permission to publicly deposit or share the raw data, as ownership and distribution rights are retained by the data provider. Data may be made available only with permission from the third‐party data provider, where applicable.
