# Supplementary material for: Microbiome‐Based Assessment of Topical Scalp Product‐Associated Microbiome Modulation
Source: J Cosmet Dermatol. 2026 Oct 28;25(11):e71238. doi: 10.1111/jocd.71238 (PMC13716926; doi:10.1111/jocd.71238)
Supplement: Supplementary file 1 [file JOCD-25-e71238-s001.zip › jocd71238-sup-0001-TablesS1-S3/Cnamnetense_prevalence_table.pdf]

Timepoint	n	n_present	Prevalence	Mean	Mean if present
T1	56	0	0.00	0.00	0.00
T2	56	0	0.00	0.00	0.00
T3	56	36	64.29	11.36	17.67
